# Supplementary material for: Mendelian randomization and colocalization analyses reveal an association between short sleep duration or morning chronotype and altered leukocyte telomere length
Source: Commun Biol. 2023 Oct 6;6:1014. doi: 10.1038/s42003-023-05397-7 (PMC10558505; doi:10.1038/s42003-023-05397-7)
Supplement: Supplementary file 2 — Supplementary Information [file 42003_2023_5397_MOESM2_ESM.pdf]

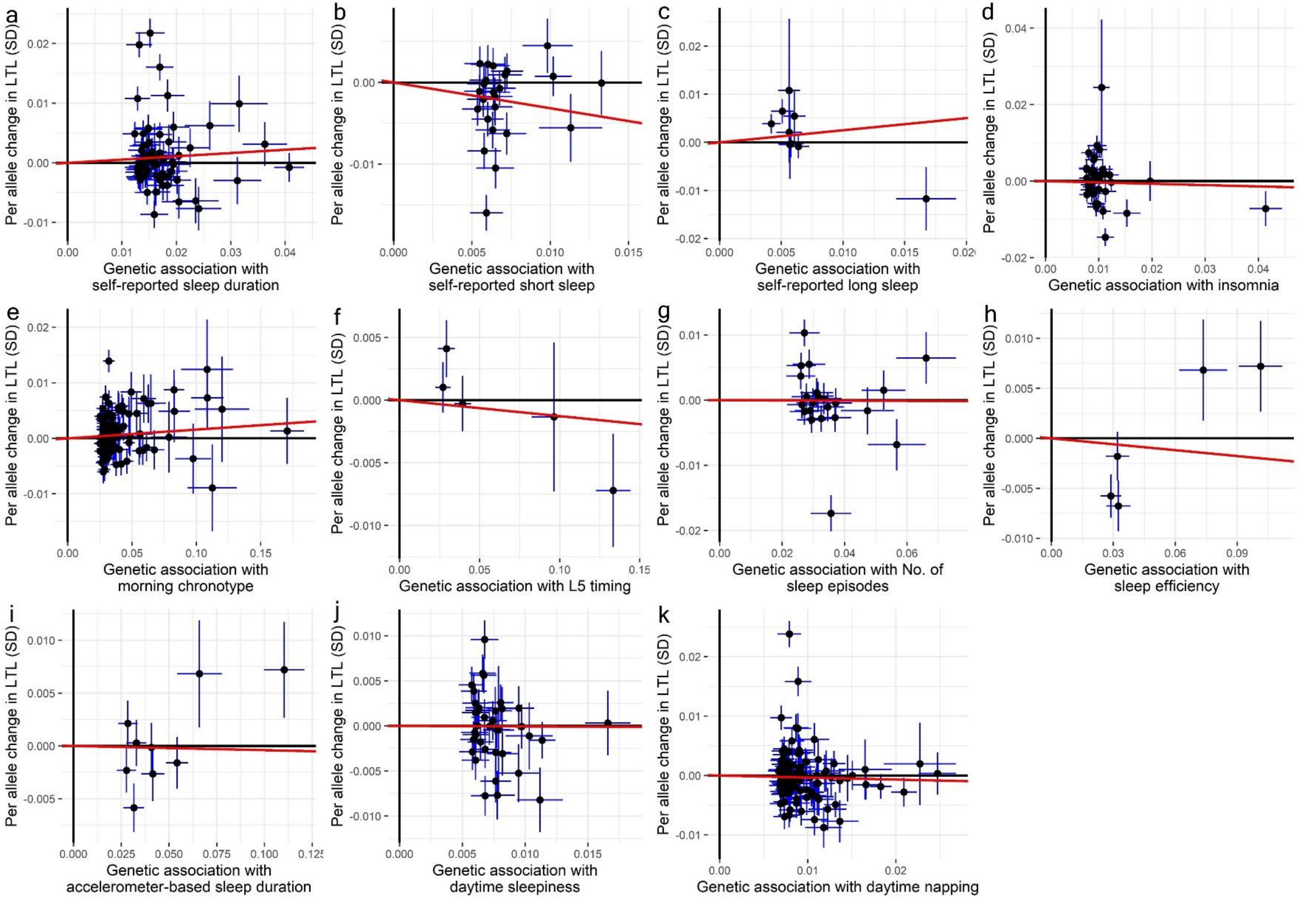

a
Per allele change in LTL (SD)
Genetic association with self-reported sleep duration
b
Per allele change in LTL (SD)
Genetic association with self-reported short sleep
c
Per allele change in LTL (SD)
Genetic association with self-reported long sleep
d
Per allele change in LTL (SD)
Genetic association with insomnia
e
Per allele change in LTL (SD)
Genetic association with morning chronotype
f
Per allele change in LTL (SD)
Genetic association with L5 timing
g
Per allele change in LTL (SD)
Genetic association with No. of sleep episodes
h
Per allele change in LTL (SD)
Genetic association with sleep efficiency
i
Per allele change in LTL (SD)
Genetic association with accelerometer-based sleep duration
j
Per allele change in LTL (SD)
Genetic association with daytime sleepiness
k
Per allele change in LTL (SD)
Genetic association with daytime napping

Supplementary Figure 1. Scatter plot of genetic association with LTL against associations with 11 sleep-related traits.

Abbreviations: L5 timing, least active 5 hours timing; LTL, leukocyte telomere length; SD, standard derivation.

The associations (*β*-coefficients) of a) self-reported sleep duration, b) self-reported short sleep, c) self-reported long sleep, d) insomnia, e) morning chronotype, f) L5 timing, g) No. of sleep episodes, h) sleep efficiency, i) accelerometer-based sleep duration, j) daytime sleepiness, and k) daytime napping on LTL were indicated by red solid lines using the inverse-variance weighted (IVW) method. Scatters showed associations of SNPs with sleep-related traits and LTL, with dotted cross-hairs indicating standard errors (SE).

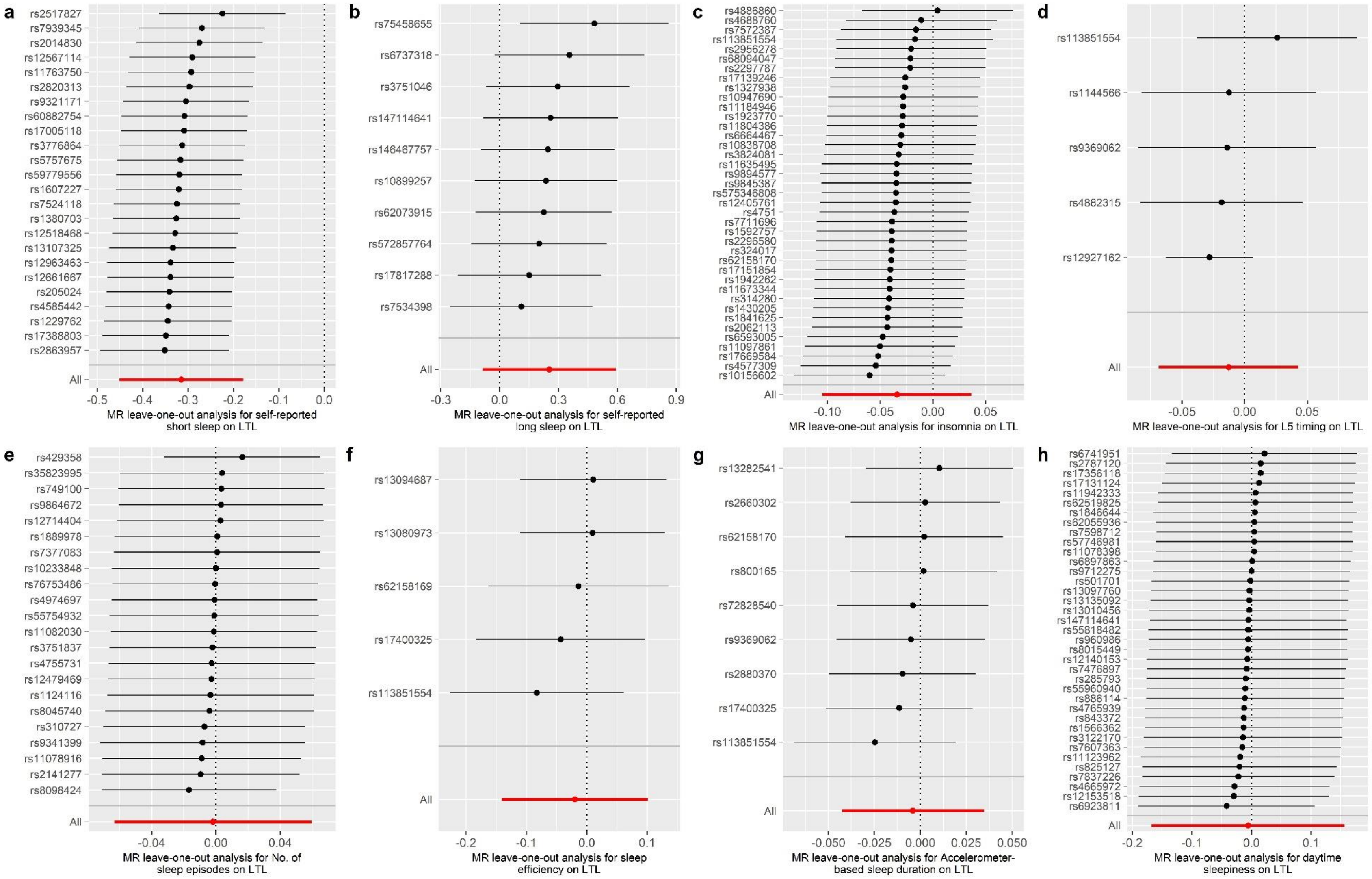

a
MR leave-one-out analysis for self-reported short sleep on LTL
b
MR leave-one-out analysis for self-reported long sleep on LTL
c
MR leave-one-out analysis for insomnia on LTL
d
MR leave-one-out analysis for L5 timing on LTL
e
MR leave-one-out analysis for No. of sleep episodes on LTL
f
MR leave-one-out analysis for sleep efficiency on LTL
g
MR leave-one-out analysis for Accelerometer-based sleep duration on LTL
h
MR leave-one-out analysis for daytime sleepiness on LTL
All

Supplementary Figure 2. Plots of leave-one-out analyses for MR analyses of 8 sleep-related traits on LTL.

Abbreviations: L5 timing, least active 5 hours timing; LTL, leukocyte telomere length.

The red lines represent the relationships of a) self-reported short sleep, b) self-reported long sleep, c) insomnia, d) L5 timing, e) No. of sleep episodes, f) sleep efficiency, g) accelerometer-based sleep duration, and h) daytime sleepiness with LTL using inverse-variance weighted (IVW) method.

The black line is the deviation of the 95% confidence interval corresponding to the estimate of the SNPs.

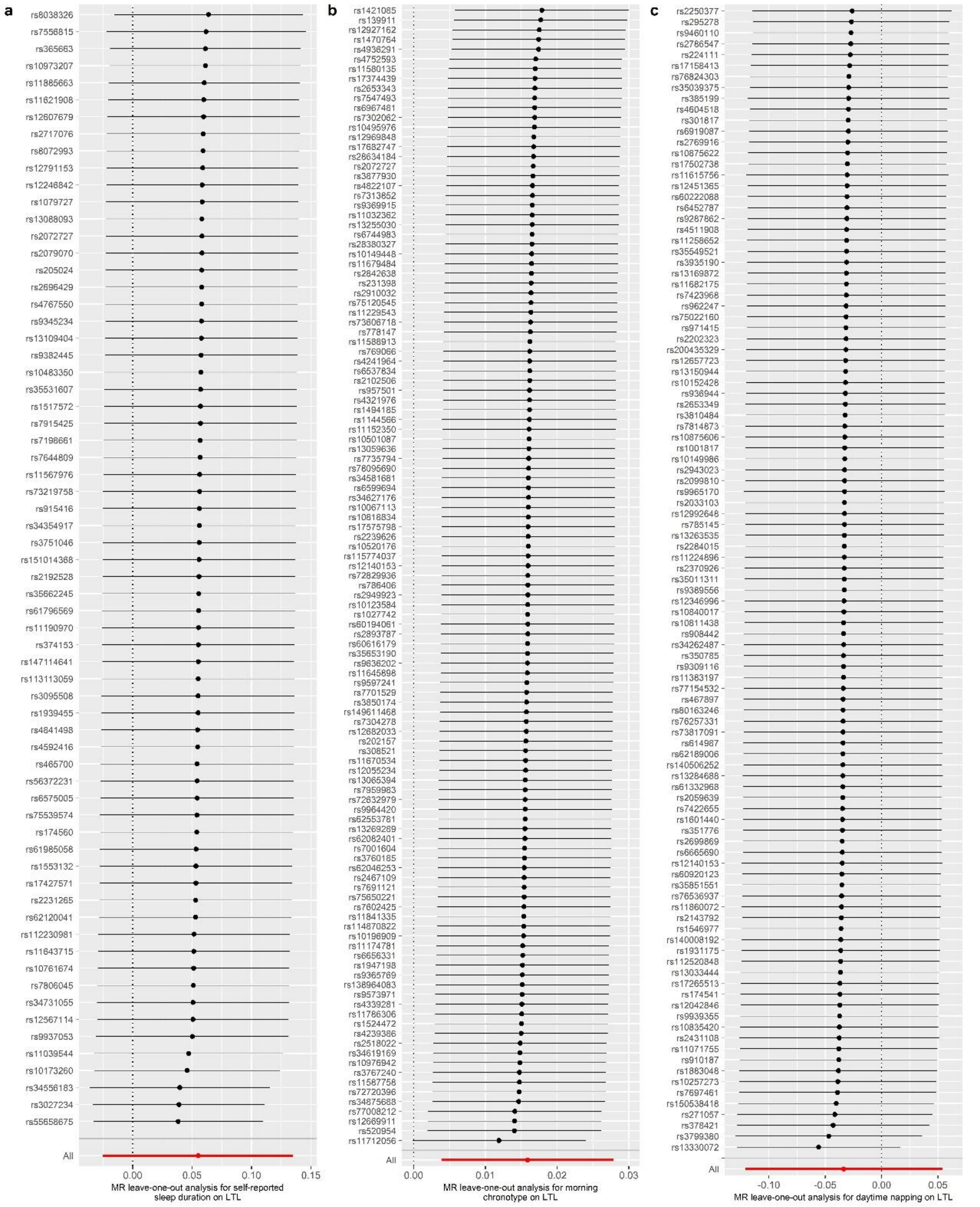

Supplementary Figure 3. Leave-one-out analysis plots for MR investigations of three other sleep-related traits on LTL, comprising self-reported sleep duration, morning chronotype, and daytime napping.

Abbreviations: LTL, leukocyte telomere length.

The red lines represent the relationships of a) self-reported sleep duration, b) morning chronotype, and c) daytime napping with LTL using inverse-variance weighted (IVW)

method.

The black line is the deviation of the 95% confidence interval corresponding to the estimate of the SNPs.

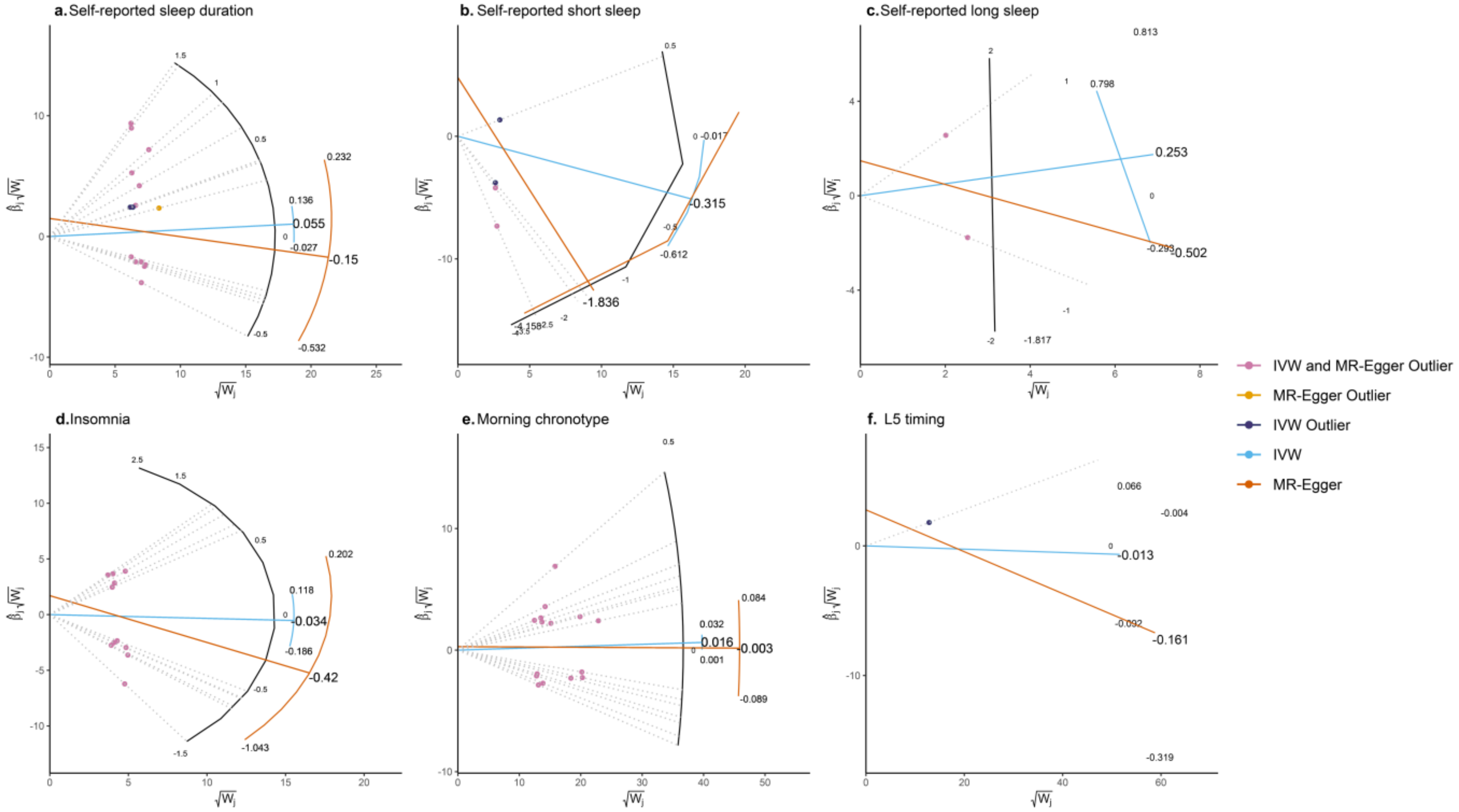

a. Self-reported sleep duration
b. Self-reported short sleep
c. Self-reported long sleep
d. Insomnia
e. Morning chronotype
f. L5 timing
0.055
-0.15
-0.315
-1.836
0.253
-0.502
-0.034
-0.42
0.016
0.003
-0.013
-0.161
IVW and MR-Egger Outlier
MR-Egger Outlier
IVW Outlier
IVW
MR-Egger

Supplementary Figure 4. Radial Mendelian randomization plots of 6 sleep-related traits on LTL.

Abbreviations: IVW, inverse-variance weighted; L5 timing, least active 5 hours timing; LTL, leukocyte telomere length.

The figure showed radial plots of a) self-reported sleep duration, b) self-reported short sleep, c) self-reported long sleep, d) insomnia, e) morning chronotype, f) L5 timing on LTL.

Radial MR method identify outliers with the most weight in the MR analysis and the largest contribution to Cochran's Q statistic (for radial IVW) or Rucker's Q statistic (for radial Egger) for heterogeneity, which may then be removed and the data re-analyzed.

Radial curve displays the ratio estimate for the outliers (identified using Radial MR), as well as the Radial IVW (in blue) and Radial MR-Egger regression (in orange).

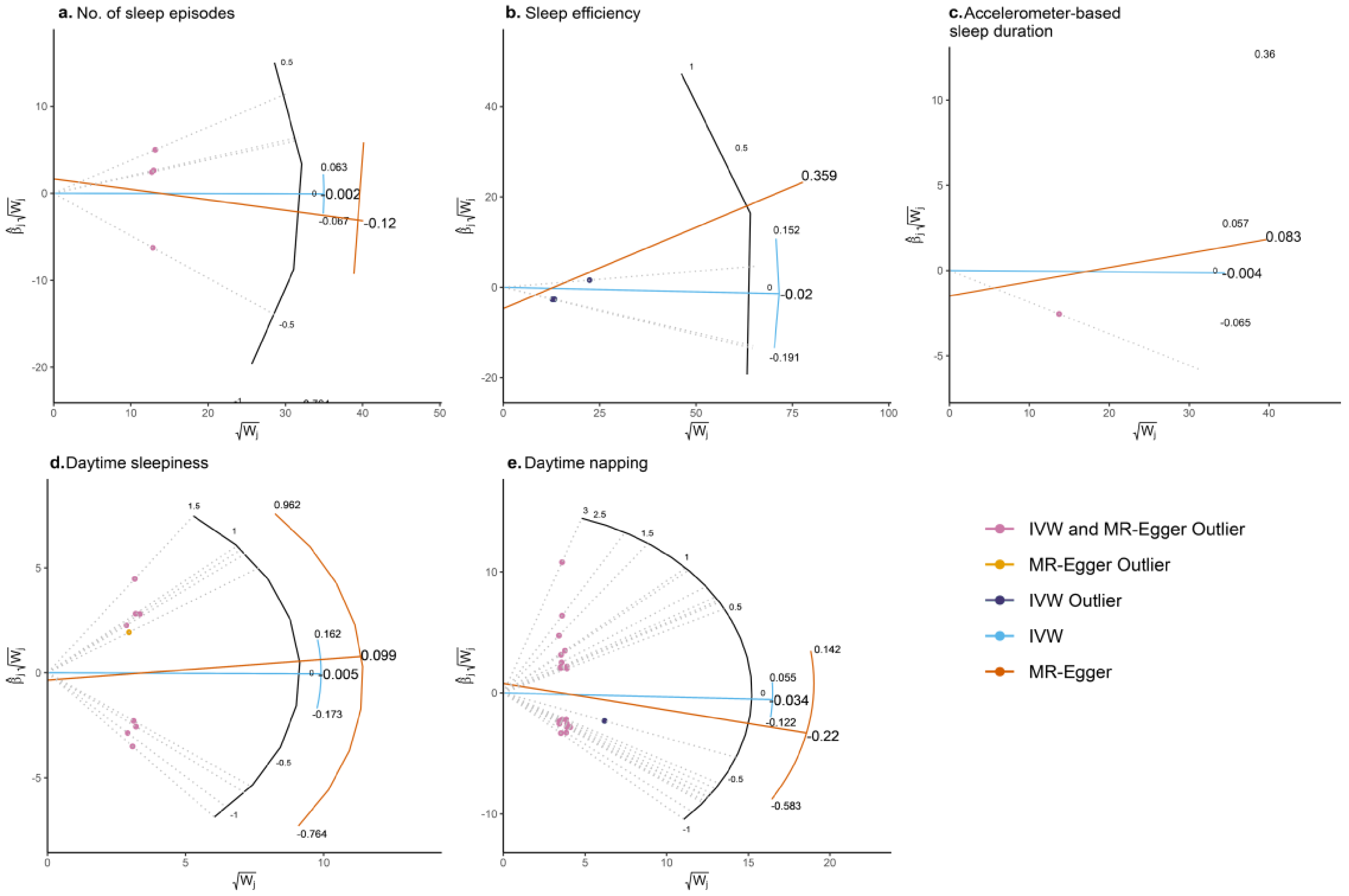

a. No. of sleep episodes
b. Sleep efficiency
c. Accelerometer-based sleep duration
d. Daytime sleepiness
e. Daytime napping
$\hat{\beta}_j\sqrt{W_j}$
$\sqrt{W_j}$
0.063
-0.002
-0.067
-0.12
0.359
0.152
-0.02
-0.191
0.36
0.057
0.083
-0.004
-0.065
0.962
0.162
0.099
0.005
-0.173
-0.764
0.142
0.055
0.034
-0.122
-0.22
-0.583
IVW and MR-Egger Outlier
MR-Egger Outlier
IVW Outlier
IVW
MR-Egger

Supplementary Figure 5. Radial Mendelian randomization plots of other 5 sleep-related traits on LTL.

Abbreviations: IVW, inverse-variance weighted; LTL, leukocyte telomere length.

The figure showed radial plots of a) No. of sleep episodes, b) sleep efficiency, c) accelerometer-based sleep duration, d) daytime sleepiness, and e) daytime napping on LTL.

Radial MR method identify outliers with the most weight in the MR analysis and the largest contribution to Cochran's Q statistic (for radial IVW) or Rucker's Q statistic (for radial Egger) for heterogeneity, which may then be removed and the data re-analyzed.

Radial curve displays the ratio estimate for the outliers (identified using Radial MR), as well as the Radial IVW (in blue) and Radial MR-Egger regression (in orange).
