# Supplementary material for: Mendelian randomization and colocalization analyses reveal an association between short sleep duration or morning chronotype and altered leukocyte telomere length
Source: Commun Biol. 2023 Oct 6;6:1014. doi: 10.1038/s42003-023-05397-7 (PMC10558505; doi:10.1038/s42003-023-05397-7)
Supplement: Supplementary file 3 — Description of Supplementary Materials [file 42003_2023_5397_MOESM3_ESM.docx]

**Description of Additional Supplementary Files**

**File name:** Supplementary Data 1

**Description:** Genetic variants for Mendelian randomization analysis of the associations between sleep-related phenotypes and leukocyte telomere length.

**File name:** Supplementary Data 2

**Description:** Strength of instrumental variables for sleep-related phenotypes.

**File name:** Supplementary Data 3

**Description:** Associations between genetically predicted sleep-related phenotypes and leukocyte telomere length.

**File name:** Supplementary Data 4

**Description:** MR results of sleep-related traits on leukocyte telomere length after removing outliers (identified using Radial MR).

**File name:** Supplementary Data 5

**Description:** Mendelian randomization results after Steiger filtering.

**File name:** Supplementary Data 6

**Description:** Associations between genetically predicted sleep-related phenotypes and leukocyte telomere length after removing pleiotropic SNPs.

**File name:** Supplementary Data 7

**Description:** Genetic variants for Mendelian randomization analysis of the relationships of leukocyte telomere length with chronotype and short sleep duration.

**File name:** Supplementary Data 8

**Description:** Associations of genetically predicted leukocyte telomere length with short sleep duration and chronotype.

**File name:** Supplementary Data 9

**Description:** Associations of genetically predicted short sleep duration with leukocyte telomere length controlled for smoking, drinking, and BMI using multivariable Mendelian randomization.

**File name:** Supplementary Data 10

**Description:** Associations of genetically predicted chronotype with leukocyte telomere length controlled for insomnia, smoking, drinking and sleep duration using multivariable Mendelian randomization.

**File name:** Supplementary Data 11

**Description:** The result from colocalization analysis of chronotype and short sleep duration with LTL.

**File name:** Supplementary Data 12

**Description:** The degree of overlapping between samples of sleep-related traits and samples of leukocyte telomere length.

**File name:** Supplementary Data 13

**Description:** MRlap estimates for the causal associations between sleep-related traits and leukocyte telomere length.
