# Supplementary material for: Mendelian randomization and colocalization analyses reveal an association between short sleep duration or morning chronotype and altered leukocyte telomere length
Source: Commun Biol. 2023 Oct 6;6:1014. doi: 10.1038/s42003-023-05397-7 (PMC10558505; doi:10.1038/s42003-023-05397-7)
Supplement: Supplementary file 5 — Reporting summary [file 42003_2023_5397_MOESM5_ESM.pdf]

nature portfolio

Corresponding author(s): Zhenqian Wang

Last updated by author(s): Sep 23, 2023

Reporting Summary

Nature Portfolio wishes to improve the reproducibility of the work that we publish. This form provides structure for consistency and transparency in reporting. For further information on Nature Portfolio policies, see our Editorial Policies and the Editorial Policy Checklist.

Statistics

For all statistical analyses, confirm that the following items are present in the figure legend, table legend, main text, or Methods section.

n/a	Confirmed	
☐	☒	The exact sample size (*n*) for each experimental group/condition, given as a discrete number and unit of measurement
☐	☒	A statement on whether measurements were taken from distinct samples or whether the same sample was measured repeatedly
☐	☒	The statistical test(s) used AND whether they are one- or two-sided<br>*Only common tests should be described solely by name; describe more complex techniques in the Methods section.*
☐	☒	A description of all covariates tested
☐	☒	A description of any assumptions or corrections, such as tests of normality and adjustment for multiple comparisons
☐	☒	A full description of the statistical parameters including central tendency (e.g. means) or other basic estimates (e.g. regression coefficient) AND variation (e.g. standard deviation) or associated estimates of uncertainty (e.g. confidence intervals)
☐	☒	For null hypothesis testing, the test statistic (e.g. *F*, *t*, *r*) with confidence intervals, effect sizes, degrees of freedom and *P* value noted<br>*Give P values as exact values whenever suitable.*
☒	☐	For Bayesian analysis, information on the choice of priors and Markov chain Monte Carlo settings
☒	☐	For hierarchical and complex designs, identification of the appropriate level for tests and full reporting of outcomes
☐	☒	Estimates of effect sizes (e.g. Cohen's *d*, Pearson's *r*), indicating how they were calculated

Our web collection on statistics for biologists contains articles on many of the points above.

Software and code

Policy information about availability of computer code

Data collection	The data used in the analysis was publicly available. Summary statistics of 11 sleep-related traits could be obtained from https://sleep.hugeamp.org/downloads.html and summary statistics of telomere length could be obtained from https://figshare.com/s/caa99dc0f76d62990195.
Data analysis	All analyses were conducted using various R software (version 4.2.2) packages including TwoSampleMR (version 0.5.6), MVMR (version 0.3), coloc (version 5.1.0.1), locuscomparer (version 1.0.0), MR-PRESSO (version 1.0), and MRlap (version 0.0.3.0), MendelianRandomization (version 0.7.0), and RadialMR (version 1.1), with a two-sided approach.

For manuscripts utilizing custom algorithms or software that are central to the research but not yet described in published literature, software must be made available to editors and reviewers. We strongly encourage code deposition in a community repository (e.g. GitHub). See the Nature Portfolio guidelines for submitting code & software for further information.

Data

Policy information about availability of data

All manuscripts must include a data availability statement. This statement should provide the following information, where applicable:

- Accession codes, unique identifiers, or web links for publicly available datasets
- A description of any restrictions on data availability
- For clinical datasets or third party data, please ensure that the statement adheres to our policy

Only publicly available data were used in this study, and data sources and handling of these data are described in the Materials and Methods and in the Supplementary Tables. Summary-level data of sleep-related traits could be obtained from https://sleep.hugeamp.org/downloads.html and summary-level data of telomere length could be obtained from https://figshare.com/s/caa99dc0f76d62990195. Further information is available from the corresponding author upon request.

Research involving human participants, their data, or biological material

Policy information about studies with human participants or human data. See also policy information about sex, gender (identity/presentation), and sexual orientation and race, ethnicity and racism.

Reporting on sex and gender	We used summary statistics from GWAS of 11 sleep-related traits and leukocyte telomere length. These GWASs were conducted in the overall population. Thus, our study is based on overall population and the findings apply to overall population.
Reporting on race, ethnicity, or other socially relevant groupings	We used summary statistics from GWAS of 11 sleep-related traits and leukocyte telomere length. These GWASs were derived from individuals of European ancestry. The current study is based on individuals of European ancestry.
Population characteristics	These summary statistics of GWASs were mainly based on UK Biobank. The UK Biobank recruited more than 500,000 participants aged from 37 to 73 (mean age 56.5 years, 45.6% men) from 22 assessment centres across England, Scotland, and Wales between 2006 and 2010.
Recruitment	Not applicable for this study which was based on summary statistics of GWAS; no recruitment was conducted in this study.
Ethics oversight	The study is an analysis using publicly available summary data that does not require ethical approval.

Note that full information on the approval of the study protocol must also be provided in the manuscript.

Field-specific reporting

Please select the one below that is the best fit for your research. If you are not sure, read the appropriate sections before making your selection.

[x] Life sciences   [ ] Behavioural & social sciences   [ ] Ecological, evolutionary & environmental sciences

For a reference copy of the document with all sections, see nature.com/documents/nr-reporting-summary-flat.pdf

Life sciences study design

All studies must disclose on these points even when the disclosure is negative.

Sample size	Sample sizes were taken from the used genome-wide association studies. Detailed information of the sample size for these GWASs were listed as below: self-reported sleep duration (N=446,118), self-reported short sleep duration (N=106,192 cases (≤6 hours) and 305,742 controls), self-reported long duration (34,184 cases (≥9 hours) and 305,742 controls), insomnia (129,270 cases (frequent insomnia) and 108,357 controls), chronotype (252,287 cases (morning chronotype) and 150,908 controls), daytime sleepiness (N=452,071), daytime napping (N=452,633), accelerometer-based sleep duration (N=84,810), L5 timing (N=85,205), sleep efficiency (N=84,810), number of sleep episodes (N=84,441), and leukocyte telomere length (N=472,174).
Data exclusions	We selected independent single nucleotide polymorphisms (SNPs) associated with the respective exposure at a genome-wide significant threshod P<5×10^(-8) and not in high linkage disequilibrium (LD) using a clumping algorithm with cut-off r2 >0.001.
Replication	Although limited by the accessibility of another independent GWAS of leukocyte telomere length, this study included a series of sensitivity analyses and showed consistent results.
Randomization	Mendelian randomization analysis uses genetic instrumental variables (i.e., SNPs) to control confounding effects and reverse causation. A conventional randomization is not performed, however, as an assumption of random genetic allocation, the method provides causal estimates.
Blinding	Since we used summary-level data, blinding was not relevant to our study.

Reporting for specific materials, systems and methods

We require information from authors about some types of materials, experimental systems and methods used in many studies. Here, indicate whether each material, system or method listed is relevant to your study. If you are not sure if a list item applies to your research, read the appropriate section before selecting a response.

Materials & experimental systems

n/a	Involved in the study
☒	☐ Antibodies
☒	☐ Eukaryotic cell lines
☒	☐ Palaeontology and archaeology
☒	☐ Animals and other organisms
☒	☐ Clinical data
☒	☐ Dual use research of concern
☒	☐ Plants

Methods

n/a	Involved in the study
☒	☐ ChIP-seq
☒	☐ Flow cytometry
☒	☐ MRI-based neuroimaging
